# Traffic-Oriented Three-Dimensional Vehicle Reconstruction Using Fixed Roadside Monocular Camera Sensors

**DOI:** 10.3390/s26041324

**Published:** 2026-02-18

**Authors:** Chu Zhang, Yuxin Zhang, Liangbin Li, Xianhua Cai

**Affiliations:** School of Transportation, Southeast University, Nanjing 211189, China; zhangchu0720@seu.edu.cn (C.Z.); 15207969776@163.com (L.L.); 101007486@seu.edu.cn (X.C.)

**Keywords:** camera-based sensing, roadside monocular cameras, vehicle 3D reconstruction, intelligent transportation systems, structure-from-motion

## Abstract

**Highlights:**

**What are the main findings?**

- A traffic-oriented framework enables reliable 3D vehicle reconstruction using fixed roadside monocular camera sensors under real-world traffic conditions.
- Joint use of semantic and non-semantic feature points significantly improves reconstruction accuracy and efficiency compared with conventional incremental SfM methods.

**What are the implications of the main findings?**

- Existing roadside camera infrastructures can be leveraged to enhance three-dimensional traffic perception without additional sensing hardware.
- The proposed approach supports scalable deployment of camera-based sensing systems for intelligent transportation applications such as traffic monitoring and analysis.

**Abstract:**

Fixed roadside monocular cameras are widely used as low-cost sensing devices in intelligent transportation systems; however, extracting reliable three-dimensional (3D) information from such sensors remains challenging due to limited baselines, long observation distances, and moving vehicles. This paper presents a traffic-oriented 3D vehicle reconstruction framework based on monocular image sequences captured by fixed roadside camera sensors. Semantic and non-semantic vehicle feature points are jointly exploited to balance structural consistency and surface completeness, and a feature-map-consistency-based optimization strategy is introduced to refine feature point localization and reduce reprojection errors. In addition, an optimized incremental Structure-from-Motion (SfM) pipeline incorporating traffic-aware initialization, keyframe selection, and local bundle adjustment is developed to improve reconstruction efficiency. Experiments on real-world traffic surveillance videos show that the proposed method reduces the mean reprojection error by 13.6% and shortens reconstruction time by 43.9% compared with widely used incremental SfM systems.

## 1. Introduction

With the rapid development of intelligent transportation systems (ITS), roadside video surveillance has become one of the most widely deployed camera-based sensing infrastructures in urban traffic networks [1]. Owing to their low installation cost, extensive spatial coverage, and continuous operation, fixed roadside surveillance cameras serve as important vision sensors for traffic monitoring, traffic flow analysis, incident detection, and infrastructure management. Compared with vehicle-mounted sensors or dedicated perception devices such as LiDAR, roadside camera sensors provide a scalable and economically viable solution for large-scale traffic perception in real-world environments.

However, the information captured by roadside surveillance cameras is inherently two-dimensional, which limits the capability of traffic systems to accurately perceive spatial structures and dynamic interactions among vehicles. Although two-dimensional video analysis techniques have achieved remarkable success in tasks such as vehicle detection, tracking, and counting, they are insufficient for applications that require spatial understanding, including precise vehicle localization, speed estimation, collision analysis, and traffic scene reconstruction. Consequently, 3D vehicle reconstruction from existing surveillance videos has attracted increasing attention as a promising approach to enhance the spatial intelligence of traffic monitoring systems without additional hardware deployment [2].

Reconstructing 3D vehicles from fixed monocular surveillance cameras, however, remains a highly challenging task. Unlike multi-view or vehicle-mounted camera scenarios, roadside surveillance systems typically suffer from limited baselines, long observation distances, and moving targets. Small localization errors in image space may lead to significant deviations in reconstructed 3D geometry, making conventional 3D reconstruction pipelines unstable or inefficient in traffic surveillance scenarios. Moreover, the dynamic nature of traffic scenes, including vehicle occlusions, illumination changes, and viewpoint variations, further complicates reliable 3D reconstruction from monocular image sequences [3].

Monocular 3D reconstruction in traffic surveillance has therefore attracted increasing interest, as fixed roadside cameras are widely deployed yet provide only single-view observations. Existing approaches can generally be categorized into learning-based methods, geometry-based methods, and hybrid pipelines that combine deep features with geometric optimization [4]. Learning-based approaches often rely on deformable templates or end-to-end neural networks trained on large-scale datasets [5]. While these methods can produce visually appealing 3D models, their performance is highly dependent on training data diversity and computational resources, which limits their applicability in real-time traffic monitoring systems [6]. In contrast, geometry-based methods, particularly those based on Structure-from-Motion (SfM), offer better interpretability and generalization ability [7]. Nevertheless, traditional SfM pipelines are not specifically designed for fixed roadside cameras and often struggle with efficiency and robustness when applied to traffic surveillance scenarios.

To address these challenges, this paper focuses on 3D vehicle reconstruction from monocular image sequences captured by fixed roadside surveillance cameras. Instead of pursuing a purely data-driven solution, we adopt a traffic-oriented system-level framework that integrates deep learning-based feature extraction with geometry-based reconstruction. By exploiting the structural characteristics of vehicles and their motion patterns in traffic scenes, the proposed method enhances both reconstruction accuracy and computational efficiency. Specifically, semantic vehicle feature points are introduced to provide structural guidance, while non-semantic feature points are utilized to ensure surface completeness. A feature-map-consistency-based optimization strategy is further employed to refine feature point localization, reducing reprojection errors caused by long-distance observation. In addition, an optimized incremental SfM pipeline tailored to traffic surveillance scenarios is designed by incorporating traffic-aware initialization, keyframe selection, and local bundle adjustment.

Through experiments on real-world traffic surveillance videos, the proposed method demonstrates superior performance in terms of reconstruction accuracy and efficiency compared with widely used incremental SfM systems. The results indicate that reliable and efficient 3D vehicle reconstruction can be achieved using existing roadside camera infrastructures, providing a practical foundation for advanced traffic monitoring and analysis applications.

## 2. Literature

### 2.1. Extraction of Feature Points

Feature point extraction is the foundation of 3D vehicle reconstruction. Harris et al. [8] used a second-moment matrix or an autocorrelation matrix to identify directions with significant changes in gray values, ensuring that corner point extraction is to some extent invariant to image rotation and illumination. However, this algorithm is relatively sensitive to scale changes. To achieve faster processing speed, several improved corner detection algorithms were subsequently developed. For example, the FAST algorithm [9] efficiently determines corner points by calculating the gray-level differences between a pixel and its neighboring pixels, although the detected corner points may not be optimal. Based on the FAST algorithm, Rublee et al. [3] combined it with the BRIEF feature descriptor algorithm [10] and proposed the ORB algorithm, which maintains high speed while incorporating orientation information and a scale pyramid to reduce the influence of noise and image transformations. Among blob detection algorithms, the classic ones include SIFT [11] and SURF [12], both of which construct scale spaces using second-order derivatives. According to existing research, corner detection algorithms offer high precision, whereas blob detection algorithms capture more image features and demonstrate better stability in complex and dynamic scenes.

Unlike traditional handcrafted feature point design, deep learning methods use data to train neural networks for end-to-end feature point extraction. DeTone et al. [4] proposed the SuperPoint network framework, which is one of the most effective deep learning approaches to date. It employs a self-supervised training strategy and further enhances the model’s generalization ability through data augmentation, enabling the detection of more robust feature points. Currently, the performance of deep learning–based feature point extraction methods has generally matched or even surpassed that of traditional methods, particularly in challenging scenarios such as weak textures and drastic illumination changes.

Despite the significant progress in both handcrafted and learning-based feature extraction methods, most existing approaches are primarily developed and evaluated under general computer vision settings. In roadside traffic surveillance scenarios, feature extraction faces additional challenges such as long observation distances, limited image resolution for target vehicles, frequent occlusions, and complex illumination variations. These factors often lead to unstable feature localization and insufficient feature density on vehicle surfaces, which may significantly degrade the performance of subsequent 3D reconstruction processes. Therefore, feature extraction strategies tailored to traffic surveillance environments are still required to improve robustness and reconstruction reliability.

### 2.2. Feature Point Matching

Feature point matching establishes correspondences between feature points in two or more images and can be categorized into traditional and deep learning–based methods. The main idea of traditional matching methods is to construct distinctive feature descriptors for feature points, measure their similarity by computing distances between descriptors, and finally eliminate mismatched points [13]. Deep learning–based matching methods optimize the traditional pipeline. Sarlin et al. [5] proposed the SuperGlue network, which introduces a flexible aggregation strategy based on graph neural networks and attention mechanisms. This approach can simultaneously perceive potential 3D scene information and perform feature matching, significantly improving matching accuracy. Inspired by SuperGlue, Sun et al. [14] developed the LoFTR algorithm, which employs a Transformer module [15] with self- and cross-attention layers to process dense local features extracted from convolutional networks. LoFTR eliminates the need for explicit feature point detection and achieves precise matching in low-texture or blurred regions. To address SuperGlue’s high computational cost, Lindenberger et al. [16] proposed LightGlue in 2023, which optimizes the network structure and introduces pruning strategies to achieve faster matching while maintaining high accuracy.

Although deep learning-based matching methods have achieved superior accuracy in challenging visual conditions, their high computational cost and dependency on large-scale training data limit their applicability in real-time traffic surveillance systems. In practical ITS deployments, feature matching algorithms are required to balance accuracy, efficiency, and scalability, especially when processing continuous video streams from large numbers of roadside cameras. Consequently, lightweight and reliable matching strategies remain essential components in traffic-oriented 3D reconstruction pipelines.

### 2.3. Vehicle 3D Reconstruction Methods

Currently, video-based 3D reconstruction of vehicles is mainly categorized into traditional and deep learning methods. The number of cameras used was further divided into monocular, binocular, and multi-camera setups. The constructed 3D vehicle models can be classified into three types: mesh, voxels, and point clouds. Additionally, some researchers use a wireframe model constructed from the main feature points of vehicles (such as wheels and lights) to represent the vehicle skeleton.

Due to their simplicity, ease of storage, and computational efficiency, wireframe models have attracted increasing research interest in recent years. Chhaya et al. [17] combined the Deformable Part Model (DPM) algorithm with the SfM algorithm using multiple images captured by a single onboard camera. This approach improved SfM’s performance in monocular setups and under vehicle specular reflection conditions, where traditional SfM often fails. However, because of the algorithm’s high computational complexity, it cannot achieve real-time 3D vehicle reconstruction. Although wireframe modeling techniques are relatively mature, vehicle wireframe models typically contain only 12 three-dimensional points, making them insufficient to accurately represent the detailed vehicle structure.

The construction of vehicle point cloud models primarily relies on the incremental SfM algorithm. The modern workflow of this algorithm was well summarized and implemented by Schönberger et al. [7], and it mainly includes steps such as feature extraction and matching, triangulation, pose estimation, and bundle adjustment (BA) optimization. Similarly, SLAM systems [18] adopt the concept of incremental SfM. Although they can reconstruct parts of a vehicle’s 3D points in real time, the resulting point clouds are often too sparse to accurately recover the vehicle’s full 3D shape.

A voxel model is composed of stacked three-dimensional pixels, and the finer the model, the greater the number of voxels required, resulting in higher memory consumption. Lu et al. [2] developed the CAROM system, which enables vehicle tracking and 3D visualization. However, this system requires multi-angle videos of the same vehicle to construct a complete and detailed voxel model. Moreover, as the voxel resolution increases, the storage space occupied by the model grows rapidly.

Similar to wireframe models, mesh models also construct a vehicle’s 3D structure using connected points and lines, but they typically employ triangles or quadrilaterals as surface elements, resulting in a more detailed representation than wireframe models. For example, Lu et al. [19] performed part-level segmentation of vehicles in single images and then used a ResNet18-based method to adjust deformable templates, reconstructing textured 3D vehicle models from images captured by a single onboard camera. Most current studies [20,21] focus on adjusting deformable mesh templates to build 3D vehicle mesh models. Although this approach can produce 3D models that align well with the vehicle shapes in images, it lacks a strong geometric foundation and interpretability, depends heavily on training data, and suffers from relatively low reconstruction efficiency.

In summary, existing vehicle 3D reconstruction methods have demonstrated promising results under controlled or multi-view conditions. However, their direct application to roadside traffic surveillance scenarios remains challenging [2]. Learning-based methods often suffer from limited generalization and high computational demands [21], while traditional geometry-based approaches are not specifically designed to handle fixed-camera settings with moving vehicles and limited baselines [17]. As a result, achieving both reconstruction accuracy and efficiency in real-world traffic environments is still an open problem. These limitations motivate the development of traffic-oriented 3D reconstruction frameworks that jointly consider vehicle structure, motion characteristics, and the operational constraints of intelligent transportation systems.

## 3. Methods

This paper addresses the current limitations of 3D reconstruction techniques for moving vehicles based on a single fixed camera’s sequence of images, which suffer from insufficient accuracy and inefficiency. It explores a reconstruction technique that takes both reconstruction efficiency and model accuracy into account. This technique can swiftly and precisely restore the three-dimensional grid model of vehicles in actual traffic scenes, satisfying applications like traffic flow statistics, collision detection, and vehicle speed measurement.

**Figure 1:**
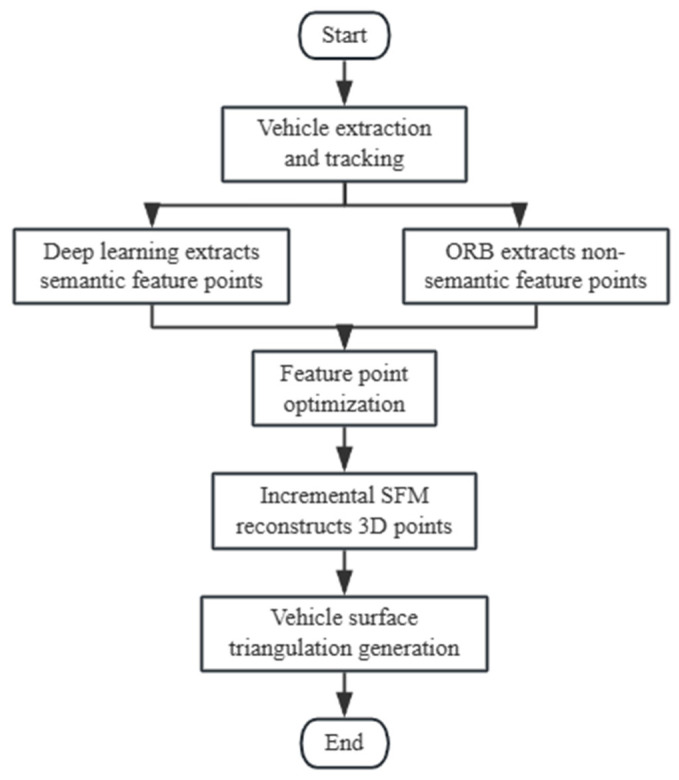
Technical roadmap.

The main components of the reconstruction method include deep learning-based vehicle semantic feature point extraction, vehicle feature point optimization, incremental SfM reconstruction of vehicle 3D points, and vehicle surface triangulation mesh generation. The steps are carried out in a sequential manner because the outcomes of the previous step must be used for the subsequent steps. The specific flow is depicted in Figure 1 below:

### 3.1. Point Extraction and Optimization

This section designs a convolutional neural network with ShuffleNetv2-50 (Table 1) as the backbone for semantic feature point extraction. ShuffleNet v2 is selected primarily for its lightweight architecture and favorable accuracy–efficiency trade-off, which is well suited for ITS deployments where roadside cameras continuously generate large-scale video streams and the reconstruction pipeline must operate under strict computational and latency constraints. Compared with heavier backbones, ShuffleNet v2 significantly reduces FLOPs and memory footprint while preserving sufficient representational capacity for vehicle semantic keypoint extraction in traffic surveillance scenarios. Moreover, the proposed framework is modular, allowing more advanced feature extraction backbones to be incorporated in future work when higher computational resources are available.

Matching is then performed based on the numbering of the semantic feature points. The idea that feature points on the same trajectory correspond to feature vectors on the feature map with invariance is used to optimize the placements of feature points. Additionally, 3D feature points that do not meet the requirements are removed.

#### 3.1.1. Extraction and Matching of Vehicle Semantic Feature Points

This study proposes an innovative approach that categorizes vehicle feature points into two distinct types: semantic feature points and non-semantic feature points, thereby facilitating the reconstruction of a more precise and structurally detailed 3D mesh model of the vehicle.

Non-semantic feature points do not convey explicit structural semantics and cannot be directly described through linguistic attributes. Instead, their spatial distribution is determined by the pixel intensity patterns across the vehicle surface, thereby reflecting the vehicle’s overall geometry. In contrast, semantic feature points encapsulate rich structural and positional information, characterized by their pixel coordinates and unique point indices, which enable accurate localization of key vehicle components. Consequently, semantic feature points serve as effective indicators of the vehicle’s primary structural characteristics.

**Figure 2:**
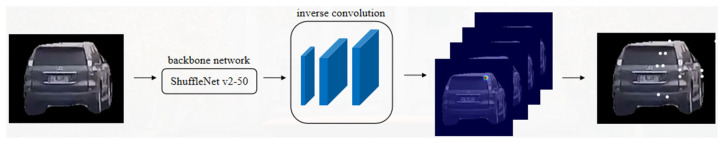
Vehicle semantic feature point extraction network structure.

In the proposed framework, semantic feature points are extracted using a convolutional neural network, whereas non-semantic feature points are detected using the conventional ORB algorithm. Specifically, a 50-layer ShuffleNetv2 backbone network is employed for semantic feature extraction. The resolution of the resulting feature maps is subsequently enhanced through three deconvolution (inverse convolution) layers, followed by fifty-four 1 × 1 convolutional kernels that generate heatmaps corresponding to 54 distinct semantic feature points. The overall network architecture is illustrated in Figure 2.

**Figure 3:**
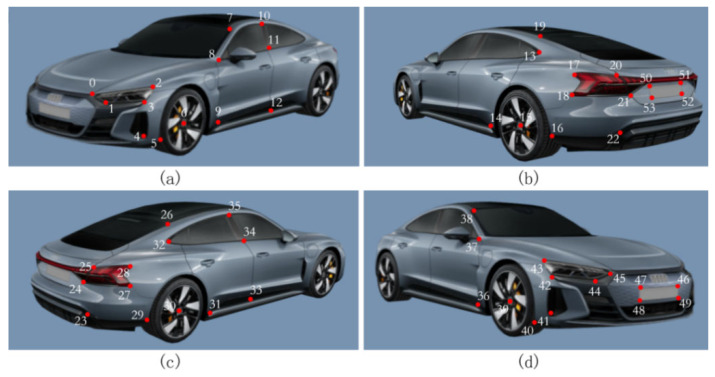
Vehicle semantic feature point distribution: (**a**) front-left view; (**b**) rear-right view; (**c**) rear-left view; (**d**) front-right view.

Using passenger vehicles as examples, Figure 3 presents the selection of 54 key semantic feature points derived from the ApolloCar3D dataset, guided by the principles of uniform spatial distribution, structural prominence, and representativeness. These feature points effectively cover the major structural components of the vehicle, and their associated attribute information—comprising pixel coordinates and point indices—enables one-to-one correspondence across multiple sequential frames of the same vehicle, ensuring consistent feature matching.

The ApolloCar3D dataset comprises 5277 images and 61,741 annotated vehicles. In addition to detailed feature point annotations, it includes CAD models with absolute dimensions for 79 vehicle categories. As the dataset primarily focuses on passenger cars, this study correspondingly emphasizes this vehicle type. Nonetheless, the proposed methodology can be readily generalized to other vehicle categories, provided that appropriately annotated datasets are available.

#### 3.1.2. Feature Point Optimization

**Figure 4:**
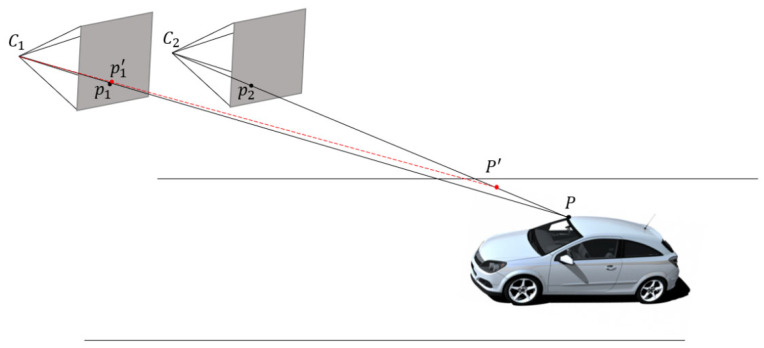
3D Feature point position error leading to 3D point offset.

In the road scenario shown in Figure 4, the motion of the vehicle-mounted camera was transformed into that of a stationary vehicle with a moving camera. Owing to the large distance between the camera and vehicle on the road, any significant errors in the positions of the corresponding feature points can lead to large offsets in the 3D points. This study proposes the use of pixel-level optimization of feature point positions based on feature maps generated by a CNN.

$C_{1}$ and $C_{2}$ denote two cameras positions; $p_{1}$ and $p_{2}$ are corresponding feature points, and P is the reconstructed 3D point. When $P_{1}$ is perturbed to $p_{1}^{\prime }$, the reconstructed 3D point shifts from P to $P^{\prime }$.

On the feature maps of various images, the feature vectors that correspond to the same feature point on the vehicle should be the same or comparable; that is, the points on the same trajectory should correspond to feature vectors with the least distances, as shown in Equation (1).

(1) $$E_{d}^{t}=\sum_{(u,v)\in t}{\left\|F_{i}^{u}-F_{j}^{v}\right\|}_{2}^{2}$$

u, v represent points on the image that belong to the trajectory t, and F is the feature vector.

**Figure 5:**
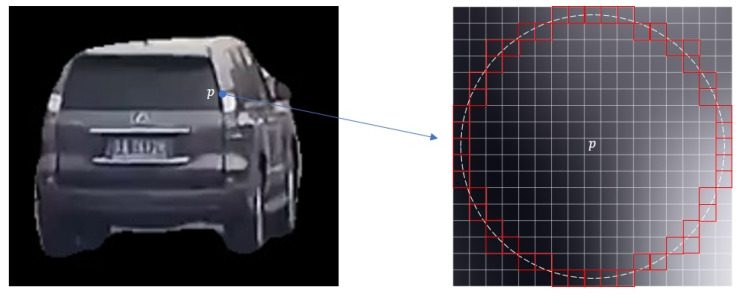
Feature point movement range. p denotes the target feature point, and the red region indicates its possible movement range.

This is a nonlinear optimization problem, which is typically solved using the Gauss–Newton method or the Levenberg–Marquardt algorithm. The Gauss–Newton method can easily fail to converge when a fixed and relatively large step size is used. Therefore, this study employs the Levenberg–Marquardt algorithm to solve the problem, with computations performed using the open-source Ceres Solver library (version 2.1.0, Google LLC, Mountain View, CA, USA). Each trajectory was optimized separately in the actual calculation to guarantee a fast optimization speed overall, and each feature point was limited to moving within an 8-pixel-radius circle (illustrated in Figure 5), thereby preventing significant drifts and limiting the range of motion of the feature points.

**Figure 6:**
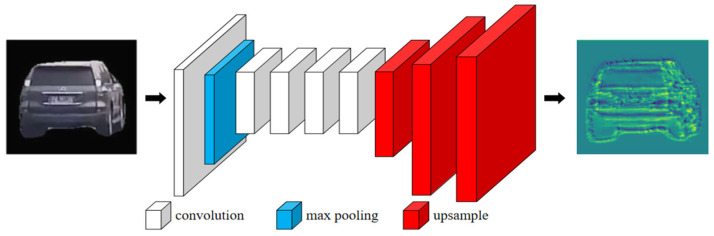
Feature map generation process. Different colors represent different processing stages in the pipeline.

In this study, the number of channels in the feature map was set to 64, while ensuring that its resolution matched that of the input image. Based on this design, the feature map output by the convolutional layer (v2) of ShuffleNet v2-50 was selected. Three upsampling layers were then applied to restore the resolution of the feature map to that of the input image. Upsampling uses the double-cubic interpolation algorithm to better handle the detailed information of the image. Figure 6 illustrates the entire feature map creation process. Each rectangle in the graphic represents a feature map; the width of the rectangle reflects the number of channels in the feature map; the result of 64 channels stacked is the final display; and different colors indicate different processes.

Once the 3D positions of the vehicle’s semantic and non-semantic feature points are calculated, it is necessary to remove the 3D spatial points that do not meet the required conditions. This ensured the accuracy of each point, resulting in a high-precision final vehicle mesh model.

The distribution of non-semantic feature points in space is primarily determined by the vehicle surface pixels. When there is a large pixel change at the location of the semantic feature point, there are more non-semantic feature points. If non-semantic feature points are found within a radius R centered on the semantic feature point, these non-semantic feature points are considered redundant and can be discarded. The value of the radius R that needs to be rejected is related to the size of the vehicle, which can be calculated using Equation (2), and α  = 0.1 was taken in this thesis.

(2) $$R=\alpha \frac{L+W+H}{3}$$

α are empirical parameters; L,W,H are the length, width and height of the vehicle, respectively.

The reprojection error reflects the accuracy of 3D points. If the average reprojection error of the 3D points exceeded three pixels, they were discarded. The average reprojection error is calculated using the following Equation (3):

(3) $$e=\sum_{j=1}^{n}{\left\|K_{j}\left[R_{j}|t_{j}\right]X-x\right\|}_{2}$$

j represents the number of keyframes involved in reconstructing point X; x is the projection of X on keyframe j; $K_{j}$ denotes the camera intrinsic parameters; $R_{j}$ is the rotation matrix; $t_{j}$ is the translation vecto.

In addition to the already reconstructed 3D points that need to be eliminated, some of the 3D points in the process of reconstruction have too low confidence level also need to be eliminated. These types of 3D points, which are observed in fewer than three consecutive keyframes, lack sufficient constraints, have low accuracy, and are likely to increase the error in subsequent iterative optimization. Therefore, they must be eliminated.

### 3.2. Reconstruction Process Optimization

Based on the temporal characteristics of road camera sequence images, this study optimizes the 3D reconstruction process using the motion laws of vehicles. It employs position optimization and bundle adjustment strategies to improve both the efficiency and accuracy of the 3D reconstruction while simultaneously constructing a detailed vehicle mesh model.

The generation of 3D points during the initialization of the reconstruction process is closely related to the selection of initial images. Therefore, an appropriate initial image must be selected based on the road scene. In this study, the frame with the largest pixel area of the vehicle in the image was selected as the first frame. The camera coordinate system corresponding to this frame was set as the world coordinate system. The camera rotation matrix for this frame was the identity matrix, and the translation vector was the zero vector. The camera pose and the 3D point coordinates for subsequent frames are referenced in the world coordinate system. The second frame must have a suitable baseline length with respect to the first frame. Because the distance between the camera and the vehicle is unknown, the actual baseline length is difficult to compute. However, the average angle between the 3D points and the optical centers of the cameras in the two frames can indirectly reflect the baseline length. In this study, the angular range between the two frames for initialization was specified as 2° to 30°. To avoid the influence of vehicle occlusion, in addition to the angular constraint, the second frame must generate at least 30 3D points from the first.

After the initialization is completed, additional video frames are required to calculate new 3D points and improve the accuracy of the already reconstructed 3D points. The SLAM-based framework was used in this study. Following reconstruction initialization, the video frames chosen for 3D point reconstruction are referred to as key frames, and they must satisfy the following criteria:

- The length of the bounding rectangle of the vehicle corresponding to the keyframe exceeds 150 pixels, and the width exceeds 120 pixels.
- The similarity with the previous keyframe is lower than 0.9. Let α be the number of feature points in the previous keyframe, and β be the number of feature points in the current video frame that match with it. The similarity between the two frames is calculated as $\frac{\alpha }{\beta }$.
- There are at least thirty car feature points shown in the key frame.

**Figure 7:**
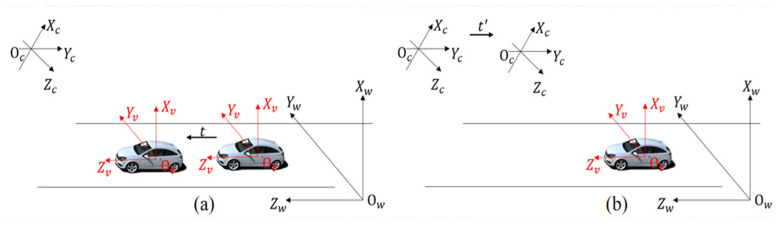
Coordinate conversion (All coordinate frames are right-handed.). (**a**) Actual scenario with a stationary camera and moving vehicle; (**b**) virtual scenario with a moving camera and stationary vehicle.

In addition, this study transforms the fixed camera and moving vehicle scenarios into a virtual scene where the camera moves and the vehicle is stationary. By calculating the camera pose, the 2D feature points of the vehicle were restored to 3D points using triangulation. Figure 7a shows the actual scenario in which the vehicle moves and the camera is stationary, whereas Figure 7b illustrates the virtual scenario in which the camera moves and the vehicle is stationary. In the figure, $O_{c}-X_{c}Y_{c}Z_{c}$, $O_{v}-X_{v}Y_{v}Z_{v}$, and $O_{w}-X_{w}Y_{w}Z_{w}$ represent the camera coordinate system, vehicle coordinate system, and world coordinate system, respectively. All coordinate frames in Figure 7 follow a consistent right-handed convention.

**Figure 8:**
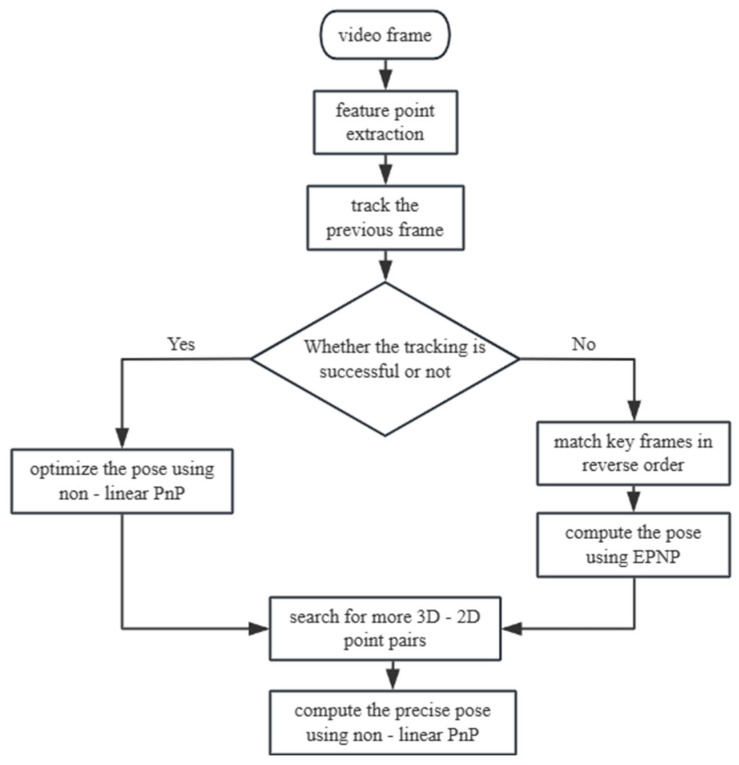
Monitoring camera position calculation flow.

The camera pose refers to the rotation matrix R and translation vector t of the camera relative to the world coordinate system. In the incremental SfM algorithm, the camera pose for each frame is obtained by feature matching, followed by Perspective-n-Point (PnP) calculation. The core idea of this algorithm is the same as that of the bundle adjustment algorithm—minimizing the reprojection error of 3D points. The difference lies in the optimization target: the nonlinear PnP algorithm optimizes only the rotation matrix and translation vector, without involving the 3D point coordinates. Because the surveillance camera captures ordered data, the SLAM temporal strategy was incorporated into the SfM to accelerate the pose estimation process. The flow is shown in Figure 8 below:

It is assumed that the camera pose of the current frame is the same as that of the previous frame. By combining the camera intrinsic parameters, the previously constructed 3D vehicle points were projected onto the current frame. If feature points in the current frame are found and successfully matched within a region containing more than 20 projected points, the camera pose of the current frame is optimized using a nonlinear PnP algorithm. If the tracking of the previous frame fails, the current frame is matched with all previous keyframes in reverse order. The keyframe with the most matched points is chosen to create a 2D-2D link with the current frame, and the feature points on this keyframe are linked to the 3D points, thereby indirectly solving the link between the 3D points and the feature points of the current frame. The positional pose of the current frame is solved using the EPnP algorithm. In addition, in the incremental SfM algorithm, the camera pose and 3D points are optimized through a global bundle adjustment after each triangulation. To reduce the computational load and speed up the reconstruction while maintaining high reconstruction accuracy, this study uses Local Bundle Adjustment (LBA) instead of the traditional Global Bundle Adjustment (GBA). After processing the vehicle video frames, a global bundle adjustment is applied to optimize the 3D points of the entire vehicle.

### 3.3. Triangular Mesh Creation on Vehicle Surfaces

After calculating the 3D points of the vehicle surface using the incremental SfM algorithm, these points must be connected according to certain rules to form a complete vehicle mesh model. This study performed triangulation on the generated 3D points to create a vehicle surface mesh. Existing algorithms for triangulating 3D points are not well-suited for handling vehicle 3D points. Therefore, this study adopts a divide-and-conquer strategy, dividing the 3D points into multiple parts and processing them individually. This approach ensured that the final vehicle surface mesh was constructed logically and highlighted the structural features of the vehicle.

Because the semantic 3D feature points are the first to be connected, the vehicle surface is divided into multiple spatial units. Each spatial unit consists of semantic 3D feature points that form a closed loop, connecting lines, and internal nonsemantic 3D feature points. Triangulation is then applied to each spatial unit to form a vehicle surface mesh. If a spatial unit cannot be closed because of missing semantic feature points, and the number of missing points is fewer than three, the missing points are supplemented by their symmetric counterparts to close the spatial unit. If no symmetric points are available for the missing points, the point is skipped, and the two adjacent points are connected to form a closed spatial unit.

To address each spatial cell, this study used a projection and dissection process. First, the best projection surface is chosen, and the 2D polygon and its internal points are triangulated. To prevent distortion, the connection between the points produced by dissection was converted to 3D space. Two categories of spatial cells were distinguished: those with and without non-semantic feature points. The former are projected and triangulated using Delaunay triangulation, which creates superior triangular meshes through edge flipping and point-by-point insertion. The latter were projected and dissected directly using auricular dissection.

Following the dissection of each spatial unit, the points create a connecting relationship that joins the 3D points in space to create a triangle mesh on the surface of the vehicle.

## 4. Experimental Results

Relevant experimental verifications were designed and conducted in this study to confirm the viability and efficacy of the proposed approach, which combines vehicle semantic feature points with non-semantic feature points, as well as the optimization strategies of the SfM algorithm. Additionally, the experimental findings were compared with those of previous studies.

### 4.1. Sets of Data

This study combined the YouTube-VIS 2021 [22], OVIS [23], and COCO2017 [24] datasets to accomplish vehicle extraction and tracking. It filters the data that contain vehicles in the YouTube-VIS 2021 and OVIS datasets and combines them with the traffic management department’s surveillance video data (the internal references of the surveillance cameras are known) to create a video dataset specifically for vehicle extraction. The ApolloCar3D dataset was presented for vehicle semantic feature point extraction to improve the accuracy of vehicle feature extraction.

All annotations of the deep learning dataset were consistently saved as structured JSON format files during data processing, and the video files were divided into individual frames and arranged neatly in the designated folders. This study uses an SQLite database for storage to efficiently handle the complex data produced by the incremental SfM algorithm during the 3D reconstruction process. The database includes several important tables, including the camera parameter table, video frame table, feature point table, two-view table, and others. The 3D mesh model of each vehicle, which is saved as a PLY format file, makes the three-dimensional structure of the vehicle visible.

### 4.2. Evaluation Metrics

The speed, accuracy, refinement, and average error of vehicle 3D model reconstruction techniques are frequently used to assess their effectiveness.

- Speed: Measured by the average time required to generate a 3D model of a vehicle.
- Level of Detail: Assessed based on the average number of 3D points and edges per vehicle.
- Accuracy: Evaluated using the 3D-PCFP (3D-Point Correspondence Feature Point) metric. This is calculated by projecting the 3D vehicle points onto a 2D image and computing the PCFP value between the projected points and their corresponding feature points.
- Average Error: Measured using the 3D-mE metric, which is the average Euclidean distance between each 3D point and its corresponding ground truth point, as shown in Equation (4).

(4) $$3D-mE=\frac{1}{P}\sum_{i=1}^{n}\sum_{j=0}^{n}{\left\|P_{ij}-p_{ij}\right\|}_{2}$$

i is the number of vehicle 3D grid models; j is the number of each point of the 3D model; $P_{ij}$ is the 3D coordinates of the corresponding point of the vehicle CAD model; $p_{ij}$ is the 3D point of the vehicle 3D mesh model; if the $p_{ij}$ is not reconstructed, then take $P_{ij}=p_{ij}=0$ is the number of points in the 3D model P is the number of all 3D points of all vehicles.

These evaluation metrics jointly reflect the geometric accuracy and structural consistency of reconstructed vehicle models, which are critical for downstream traffic applications such as vehicle localization, speed estimation, and collision analysis.

### 4.3. Results of the Experiment

This study addresses five modifications to openMVG: feature point optimization, reconstruction initialization, keyframe selection, camera position calculation optimization, and cluster adjustment tuning optimization. COLMAP and openMVG are selected as baseline methods because they represent widely used and well-established incremental Structure-from-Motion systems in engineering applications. These systems do not rely on large-scale training data and are commonly adopted in practical reconstruction tasks, making them appropriate baselines for evaluating improvements in traffic surveillance scenarios. The enhanced openMVG was evaluated against the original openMVG and COLMAP incremental SfM reconstruction systems across many metrics. Given that openMVG and COLMAP are offline 3D reconstruction systems, a 10 s video was derived from 50 frames at 0.2 s intervals. The vehicles present in each frame were identified, and those corresponding to fewer than five frames were discarded, resulting in 13 vehicles and their respective image sequences. All three methods utilize ORB to extract vehicle features and employ reprojection error as the accuracy metric for creating 3D points; the indices of the three systems are presented in Table 2.

Comparisons with the original openMVG and COLMAP systems reveal that the optimization method proposed in this paper improves both the speed and accuracy of incremental SfM for 3D vehicle reconstruction. To ensure reconstruction accuracy, points exhibiting large reprojection errors and low confidence were filtered out. Specifically, regarding the confidence criterion: for image pairs generated by vehicle extraction and tracking with a 1 s interval, matches are considered to have low confidence if the ratio $d_{1}/d_{2}$ exceeds 0.8 (where $d_{1}$ and $d_{2}$ represent the distances to the nearest and second-nearest descriptor neighbors in the second image, respectively), calculated prior to RANSAC outlier removal. Consequently, the optimized openMVG system generates a slightly lower average number of 3D points compared to the original system.

3D mesh models for 15 cars containing CAD models from the ApolloCar3D dataset were also reconstructed. Ablation experiments were conducted to assess the impact of each optimization strategy on various metrics of the overall 3D reconstruction. The results are shown in Table 3. The √ in the table indicates that an optimization approach was employed.

**Figure 9:**
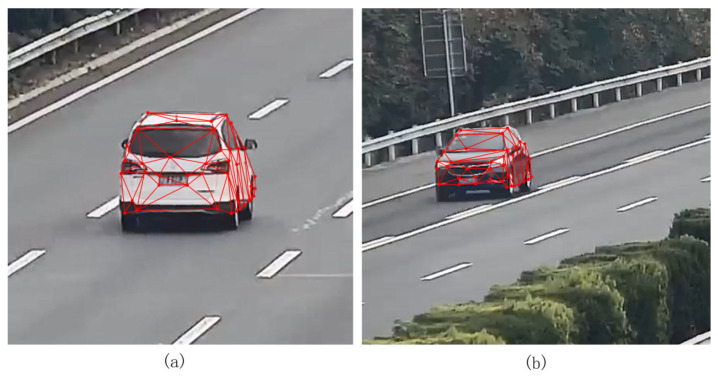
Vehicle 3D mesh model projection to the original image. (**a**) projection result at a closer distance; (**b**) projection result at a longer distance.

From the ablation experiments, it was found that after the three optimization strategies, all metrics were optimal, except the average reconstruction time. Among them, the feature point optimization strategy reduces the average error by 9.2 cm and improves the 3D point accuracy by 10.3% while consuming 0.9 s more. Positional optimization reduced the reconstruction time by 2 s while maintaining accuracy. The cluster adjustment optimization had an effect on both the mean reconstruction time and the mean error, reducing the mean reconstruction time by 1.2 s and the mean error by 3.8 cm. This improvement in computational efficiency is particularly important for intelligent transportation systems, where roadside cameras continuously generate large volumes of video data, and timely processing is required for traffic monitoring and analysis. Each optimization strategy had a small impact on the average number of 3D points generated. The 3D mesh model of the vehicle generated in this study was projected onto the original image, and the results shown in Figure 9 were obtained.

## 5. Conclusions

The main contributions of this work are summarized as follows:

(1) A traffic-oriented, engineering-oriented pipeline for 3D vehicle reconstruction from fixed roadside monocular cameras is presented, aiming at robust deployment under real-world traffic constraints.
(2) A joint use of semantic and non-semantic feature points is adopted to improve structural consistency and surface completeness, and a feature-map-consistency refinement is integrated to mitigate localization noise.
(3) Incremental SfM is optimized for traffic sequences through initialization, keyframe selection, and local bundle adjustment, improving efficiency and reducing reprojection errors.

## 6. Limitations and Future Work

Despite the encouraging results, several limitations should be acknowledged. First, the current evaluation is conducted on a limited number of vehicles and sequences, and scenario diversity (e.g., viewpoint changes, illumination variations, and occlusions) is not yet fully covered. Second, the comparisons mainly focus on classical incremental SfM systems; evaluations against specialized traffic-oriented methods and recent learning-based or hybrid pipelines are left for future work. Third, reconstruction robustness under challenging real-world conditions—such as nighttime scenes and adverse weather (rain, fog, snow), which often degrade surveillance video quality—has not been systematically quantified. In future work, we will expand the dataset with more diverse traffic environments and vehicle types, incorporate additional baselines, and explore strategies to improve robustness under low-quality surveillance data. In addition, future work will investigate multi-modal sensing by integrating complementary roadside sensing technologies to further enhance robustness in complex environments [25]. More broadly, the proposed framework may contribute to spatial sensing applications beyond traffic, including geosensing and environmental monitoring, by enabling scalable extraction of 3D information from widely deployed infrastructure sensors [26].

## Figures and Tables

**Table 1:** Structure of shuffleNetv2-50.

Layer Name	Output Size	Kernel Size	Stride	Repeat	Output Channels
Image	384×384				3
Conv v1	192×192	3×3	2	1	24
Max Pooling	96×96	3×3	2	1	24
Conv v2	48×48	$\left[\begin{array}{c} 1\times 1\\ 3\times 3\\ 1\times 1 \end{array}\right]$	2 for the first group, 1 for the rest	4	64
Conv v3	24×24	$\left[\begin{array}{c} 1\times 1\\ 3\times 3\\ 1\times 1 \end{array}\right]$	2 for the first group, 1 for the rest	8	348
Conv v4	12×12	$\left[\begin{array}{c} 1\times 1\\ 3\times 3\\ 1\times 1 \end{array}\right]$	2 for the first group, 1 for the rest	4	696
Conv v5	6×6	1×1	1	1	1024

**Table 2:** Comparison of incremental SfM systems for 3D reconstruction.

Systems	Average Number of 3D Points	Average Reconstruction Time (s)	Mean Reprojection Error (Pixels)
COLMAP	24.3	4.9	0.68
openMVG	37.1	5.7	0.59
openMVG (optimized)	32.6	3.2	0.51

**Table 3:** Ablation experiments.

Feature Point Optimization	Posture Optimization	Cluster Tuning Optimization	3D-PCFP	Average Number of 3D Points	Average Reconstruction Time (s)	3D-mE (cm)
√	√	√	95.6	39.7	5.6	18.3
	√	√	85.3	37.6	4.7	27.5
√		√	94.7	38.4	7.6	19.8
√	√		91.3	38.9	6.8	22.1

## Data Availability

The data presented in this study are available on request from the corresponding author.
